# Supplementary material for: Causal Relationship between Aging and Anorexia Nervosa: A White-Matter-Microstructure-Mediated Mendelian Randomization Analysis
Source: Biomedicines. 2024 Aug 16;12(8):1874. doi: 10.3390/biomedicines12081874 (PMC11351342; doi:10.3390/biomedicines12081874)
Supplement: Supplementary file 1 [file biomedicines-12-01874-s001.zip › Supplementary Figure/Figure S4/Singe SNP plot of MR analysis for TL to ubm-b-2074.pdf]

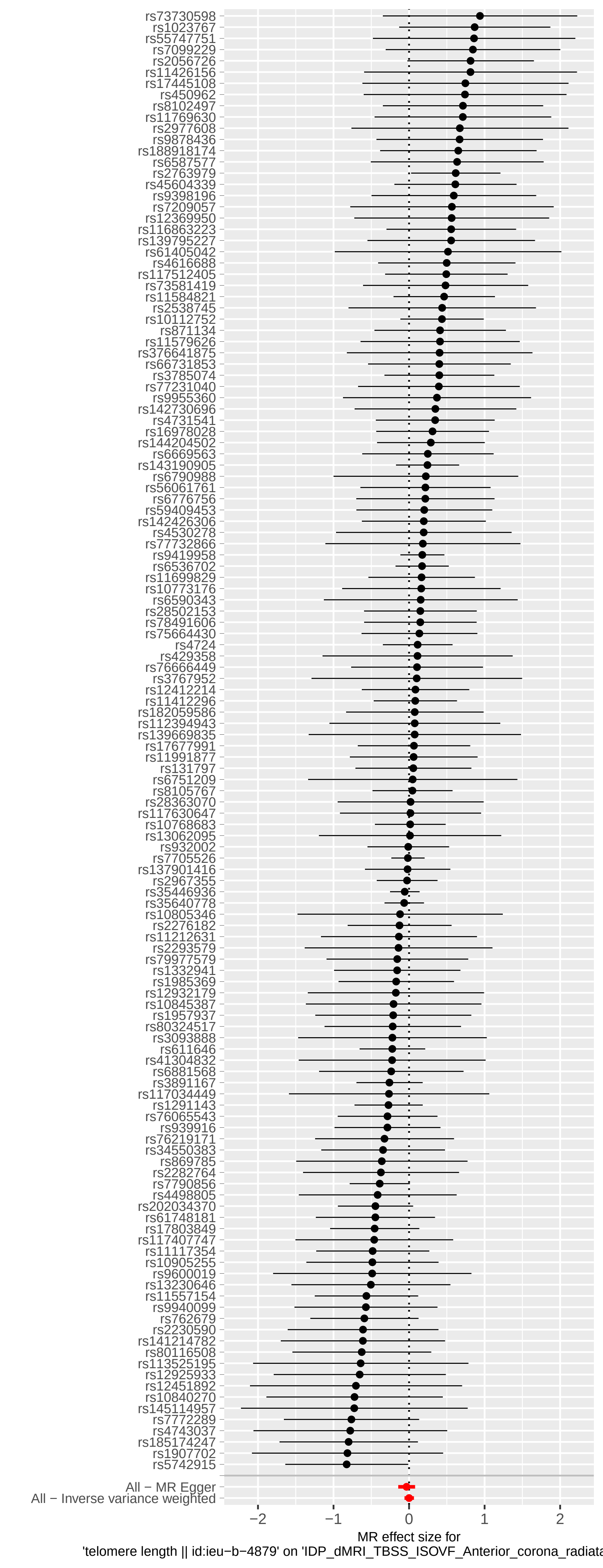
rs73730598
rs1023767
rs55747751
rs7099229
rs2056726
rs11426156
rs17445108
rs450962
rs8102497
rs11769630
rs2977608
rs9878436
rs188918174
rs6587577
rs2763979
rs45604339
rs9398196
rs7209057
rs12369950
rs116863223
rs139795227
rs61405042
rs4616688
rs117512405
rs73581419
rs11584821
rs2538745
rs10112752
rs871134
rs11579626
rs376641875
rs66731853
rs3785074
rs77231040
rs9955360
rs142730696
rs4731541
rs16978028
rs144204502
rs6669563
rs143190905
rs6790988
rs56061761
rs6776756
rs59409453
rs142426306
rs4530278
rs77732866
rs9419958
rs6536702
rs11699829
rs10773176
rs6590343
rs28502153
rs78491606
rs75664430
rs4724
rs429358
rs76666449
rs3767952
rs12412214
rs11412296
rs182059586
rs112394943
rs139669835
rs17677991
rs11991877
rs131797
rs6751209
rs8105767
rs28363070
rs117630647
rs10768683
rs13062095
rs932002
rs7705526
rs137901416
rs2967355
rs35446936
rs35640778
rs10805346
rs2276182
rs11212631
rs2293579
rs79977579
rs1332941
rs1985369
rs12932179
rs10845387
rs1957937
rs80324517
rs3093888
rs611646
rs41304832
rs6881568
rs3891167
rs117034449
rs1291143
rs76065543
rs939916
rs76219171
rs34550383
rs869785
rs2282764
rs7790856
rs4498805
rs202034370
rs61748181
rs17803849
rs117407747
rs11117354
rs10905255
rs9600019
rs13230646
rs11557154
rs9940099
rs762679
rs2230590
rs141214782
rs80116508
rs113525195
rs12925933
rs12451892
rs10840270
rs145114957
rs7772289
rs4743037
rs185174247
rs1907702
rs5742915
All − MR Egger
All − Inverse variance weighted
−2
−1
0
1
2
MR effect size for
'telomere length || id:ieu−b−4879' on 'IDP_dMRI_TBSS_ISOVF_Anterior_corona_radiata
